# Expectations and Decisions in the Volunteer’s Dilemma: Effects of Social Distance and Social Projection

**DOI:** 10.3389/fpsyg.2016.01909

**Published:** 2016-12-06

**Authors:** Joachim I. Krueger, Johannes Ullrich, Leonard J. Chen

**Affiliations:** ^1^Department of Cognitive, Linguistic, and Psychological Sciences, Brown University, ProvidenceRI, USA; ^2^Department of Psychology, University of ZurichZurich, Switzerland; ^3^Public Service DivisionSingapore, Singapore

**Keywords:** social dilemma, prosociality, expectation, rationality

## Abstract

In a *Volunteer’s Dilemma* (VoD) one individual needs to bear a cost so that a public good can be provided. Expectations regarding what others will do play a critical role because they would ideally be negatively correlated with own decisions; yet, a social-projection heuristic generates positive correlations. In a series of 2-person-dilemma studies with over 1,000 participants, we find that expectations are indeed correlated with own choice, and that people tend to volunteer more than game-theoretic benchmarks and their own expectations would allow. We also find strong evidence for a social-distance heuristic, according to which a person’s own probability to volunteer and the expectation that others will volunteer decrease as others become socially more remote. Experimentally induced expectations make opposite behavior more likely, but respondents underweight these expectations. As a result, there is a small but systematic effect of over-volunteering among psychologically close individuals.

## Introduction

“That love as such may be unable to settle a conflict can be shown by considering a harmless test case, which may pass as representative of more serious ones. Tom likes the theater and Dick likes dancing. Tom lovingly insists on going to a dance while Dick wants for Tom’s sake to go to the theater. This conflict cannot be settled by love; rather, the greater the love, the stronger will be the conflict. There are only two solutions; one is the use of emotion, and ultimately of violence, and the other is the use of reason, of impartiality, of reasonable compromise.”

Sir Karl Popper (1945/2011, p. 441)

Surviving and flourishing in the natural and the cultural world requires decision-making skills. In games against nature, humans and other animals seek to do whatever ensures the survival of their physical selves and the genes they carry (Buss, 1999). They need to forage efficiently in environments characterized by uncertainty, scarcity, and an indifference to their welfare. In social games, which often involve self-interested and only sometimes empathic conspecifics, humans need to predict what these others will do when they know that these others are also trying to figure out what they themselves will do (Hoffrage and Hertwig, 2012). Social games demand the kind of strategic reasoning that generates and makes use of expectations in a dynamical way. These games demand – as Popper realized – reason, impartiality, and compromise.

What sort of reason is it? Game theory offers a formal paradigm for the description of social games or dilemmas and for derivations of rational choice (Von Neumann and Morgenstern, 1947; Luce and Raiffa, 1957; Binmore, 2007). Orthodox game theory does not face the problem of expectation squarely; it finesses the problem of other minds by defining it away. Consider game-theory’s iconic game, the prisoner’s dilemma, or PD. The person (or ‘agent’ or ‘player’) who is rational in the game-theoretic sense defects, hoping perhaps – though not expecting – that others will cooperate. This player recognizes defection as the dominating strategy. Whatever the other player (in a 2-person game) does, this player fares better defecting. Unilateral defection pays more (or penalizes less) than bilateral cooperation, and bilateral defection pays more than unilateral cooperation. To find the rational response, the player only needs to subtract one payoff from another, do this twice, and note that the ordinal result is the same. In other words, the player only needs to understand that defection is the “sure thing” (Tversky and Shafir, 1992). As the direction of the difference is the same regardless of the expected probability of the other player cooperating (or defecting), the concept of expectation drops out.

Noting the psychological barrenness of classic game theory and worrying about its limited descriptive success (i.e., the finding that many reasonable people cooperate in the PD), revisionist theorists have reintroduced expectations as a necessary determinant of rational choice (Pruitt and Kimmel, 1977; Monterosso and Ainslee, 2003; Rapoport, 2003). Research has shown that many individuals cooperate on the condition that there is evidence or a good expectation that the other person will also cooperate (Gintis, 2000; Fischbacher et al., 2001; Nielsen et al., 2014).

A related line of research suggests that many individuals expect others to choose the same strategy that they themselves will choose, and that they therefore end up choosing cooperation (Fischer, 2009; Krueger, 2013, 2014). According to this alternative perspective on social dilemmas, the generation of behavioral expectations and their effects on own choice is neither unnecessary nor irrational. Since the days of Pascal (1995/1669) and Bernoulli (1954/1738), the multiplicative integration of expectations and values (i.e., payoffs) lies at the heart of most theories of rational choice (e.g., Ajzen and Fishbein, 2008). These theories assume that people are either able to multiply and that they choose well, or that at least their choices fit the predictions made from explicit multiplications of expectations and values, that is, people act at least *as if* they were making the requisite calculations (Berg and Gigerenzer, 2010).

The research we report in this article is concerned with the volunteer’s dilemma, or VoD, which belongs to a class of games in which rational agents would wish to choose opposite strategies. These dilemmas are known as anti-coordination games. Here each player’s goal is to mismatch the other player’s strategy, which raises particular psychological challenges (Abele et al., 2014). As in other social dilemmas (including the PD), there is a choice between one strategy that favors the self and another strategy that favors the other person or the group (Archetti and Scheuring, 2011). The outcome depends both on one’s own choice and the choice of the other, and there is an inequality: the individual and the collective outcome of mutual cooperation are better than the outcome of mutual defection (Dawes, 1980; Krueger et al., 2016). Yet, there is an incentive to defect, which raises the specter of the destructive outcome of mutual defection (Hardin, 1968). Whereas the structure of the PD makes it easy for the game-theoretic rationalist to understand that defection dominates cooperation, the VoD offers no dominating strategy. This feature is a definitional property of games that yield best results when the two agents choose different strategies, such as the game of chicken (Rapoport and Chammah, 1966, which is also know as the hawk-dove game, or its multi-player extension, the crowding game; Alpern and Reyniers, 2001). Game theory responds to this challenge with the concept of the mixed-strategy Nash equilibrium, which is designed to withhold from the other person any incentive to change strategy. Again, expectations are unnecessary for the derivation of the Nash equilibrium strategy.

**FIGURE 1:**
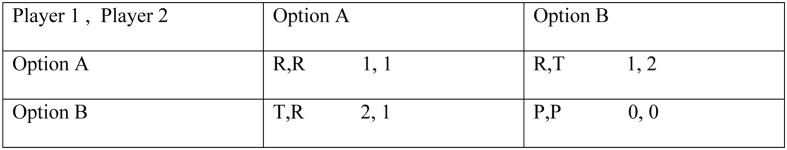
**Payoff Matrix of the Volunteer’s Dilemma.** Option A is to volunteer; Option B is to abstain.

Consider the structure of the VoD as displayed in **Figure 1**. Volunteering yields the outcome (or payoff) “R,” which stands for “Reward” (after Rapoport, 1967). R is obtained regardless of the other player’s choice. Defection yields payoff “T” (for “Temptation”) if the other player volunteers, but payoff “P” (“Penalty”) if the other defects. There is a social dilemma because T > R > P. Situations satisfying the definition of the VoD crop up throughout social life whenever a division of labor and responsibility is not regulated by contract or custom. Lecturers, for example, hope for a student to volunteer to speak in class and thereby ignite discussion; victims of emergency hope that one person will help; soldiers on the battlefield sometimes need one comrade who will accept the riskiest mission so that the others may live.

When communication and coordination are impossible, each individual must decide independently what to do. Diekmann (1985) derived the mixed-strategy Nash equilibrium probability of volunteering as (R - P)/(T - P). The difference R - P can be thought of as the psychic benefit of volunteering, but also as the potential cost of not volunteering. The difference T - P represents the total cost of mutual defection, which is the sum of T - R (i.e., the temptation to defect) and R - P. We consider it psychologically implausible that people approach a social dilemma without wondering what other individuals will do. Even an orthodox game theorist assumes (or expects) that a Nash-playing person will assume that other individuals will do likewise. This is a non-trivial expectation because even though deviating from Nash cannot improve one’s own payoffs, it can hurt the payoffs of the other (Krueger et al., 2016, unpublished). In short, the game-theoretic approach postulates the belief in common knowledge, which is tantamount to a multi-level shared expectation (Thomas et al., 2014). Game theory assumes that players are not motivated by malice and that they do not expect others to be so motivated.

### Expectations: The Social Projection Hypothesis

The questions of whether people form expectations about others in social dilemmas and whether such expectations affect strategic decisions are separable. With regard to the first question, there is empirical support for the idea that people form expectations projectively: they think that others are likely to choose whichever strategy they themselves prefer. Dawes et al. (1977) presented evidence for this hypothesis (see also Messé and Sivacek, 1979) and Dawes (1989) derived a Bayesian rationale for why people *should* use their own strategic choice as a projective cue to predict the choices of others, and proved by backward induction that even a sample of one ought not be ignored lest a sample of any size would have to be ignored. This logic is particularly compelling in an information-poor environment such as an anonymous one-shot social dilemma.

With regard to the second question, it has been argued that once projection is admitted as a judgment heuristic, it cannot be ignored as a decision heuristic (Krueger and Acevedo, 2005). In the PD, for example, the rational expectation that most others – by definition – are more likely to make the same instead of a different choice will leave a person caught between the prospects of mutual cooperation and mutual defection. Being able to only predict mutuality by using the projection heuristic, a self-interested player has no reason *not* to choose cooperation. Choosing cooperation does not imply a magical belief that the other person’s behavior can be influenced but simply reflects respect for the statistical rule that one’s own choice is diagnostic of the choices of most others (Krueger, 2013; Krueger and Acevedo, 2005; Krueger et al., 2012). Social projection is beneficial in the PD because mutual cooperation is best for both the individual and the group, whereas in the VoD, projection is problematic because mutual cooperation (2R) is worse than unilateral cooperation (T+R). Ideally, a player would choose whichever strategy the other player is not choosing. If Tom knows that Dick volunteers, Tom defects. If Tom knows that Dick defects, Tom volunteers. The structure of the VoD thus challenges the human tendency to project. A player who volunteers and then estimates that the other player will also volunteer will be dissatisfied with the prospect of mutual, that is, inefficient, volunteering. A player who defects and then estimates that the other player will also defect will be unhappy with the prospect of mutual loss. In other words, these players find themselves in Popper’s dilemma of love.

If the VoD does not reward social projection, one might think that projection is low or even reversed in this dilemma. Our working hypothesis, however, is that projection will be strong nonetheless. We draw this hypothesis from past research, which has shown that projection is a reliable social heuristic even under conditions discouraging its use (Krueger and Clement, 1994; Krueger, 2003). We predict that in the VoD players’ strategy choices will be positively correlated with the choices expected of others.^1^

### Evolution: The Social Distance Hypothesis

Classic game theory is not concerned with individual differences, identity, or social categories. The theory does not simply happen to ignore such variables. Its axioms affirm their irrelevance. There is only one standard of rational choice, and everyone is assumed to meet it. In contrast, social psychology and evolutionary psychology recognize the relevance of prosocial motives and how these motives are differentially activated by the nature of the relationships between or among actors (Murphy and Ackermann, 2014; Kurzban et al., 2015). The broadest generalization emerging from theory and data is that the probability of prosocial choice decreases with social (or psychological or genetic) distance. Hamilton’s (1964) theory of inclusive fitness provides an elegant Darwinian rationale. Assuming that the survival of genes is the ultimate adaptive coin, organisms will make sacrifices if and only if the net effect on the survival of their genes is positive. Prosocial behavior will therefore decrease as the beneficiaries of these sacrifices become biologically more distant. In a classic study, Burnstein et al. (1994) showed that people come to the aid of close over distant kin in hypothetical life-and-death scenarios, whereas less serious contexts activate social norms concerning need and deservingness. Genetic relatedness is difficult to display and assess, and humans and other animals have evolved a range of cues to honestly or deceptively signal relatedness (Dawkins, 1976). Perhaps the crudest way to differentiate between close and distant others is to categorize them into ingroups and outgroups. The general finding is that people like their ingroups more than outgroups (Krueger and DiDonato, 2008), describe them in more favorable terms, and – importantly – are more willing to help ingroup than outgroup members in need (Rabbie and Horwitz, 1969; Tajfel and Turner, 1979; DiDonato et al., 2011).

From the perspective of biology, anthropology, and psychology, “bounded prosociality” is a stylized fact (De Dreu et al., 2015). Its robustness presents a challenge to traditional game theory. There is much evidence to show that people cooperate more readily with presumed ingroup members than outgroup members in a variety of social dilemmas (Balliet et al., 2014). Importantly, the increased willingness to cooperate in the context of “parochial morality” comes with the expectation that ingroup members, but not outgroup members, will also cooperate (Yamagishi and Kiyonari, 2000; Brewer, 2008). In other words, differential projection (Robbins and Krueger, 2005) tends to be accurate. Extrapolating from this research, we hypothesize that people’s readiness to volunteer and their expectations that others will volunteer both diminish over social distance. Although such a decline runs counter to the precepts of traditional game theory, it is consistent with certain social preference models of interdependent behavior (e.g., Van Lange, 1999; Fehr et al., 2005; Archetti, 2009).

Archetti (2009) developed a social-preference model to quantitatively predict the probability of volunteering for degrees of social distance. With our payoff notation, Archetti’s (2009, p. 476) equation becomes $p_{\text{v}}=1-\frac{T-R}{(T-P)\cdot \left[1+\left(1-d\right)\right]}$. The probability of volunteering, p_v_, increases as the temptation to defect, T - R, or the cost of mutual defection, T - P, decrease and as social distance, d, increases. The parameter d captures the idea that the utility of volunteering is high to the extent that the other person is socially or genetically close to the self. Consider the payoffs in **Figure 1**, namely T = 2, R = 1, and P = 0. For maximum distance (d = 1), we find that p_v_ = 0.5, which is the conventional Nash equilibrium. Neither orthodox game theory nor a biologically informed social-preference theory would assume a probability of volunteering below this benchmark.^2^ For zero distance p_v_ = 0.75. Here, the player weights the outcomes of the other as much as his or her own outcomes, and if both players do this, the sum of their outcomes is maximized. Note, however, that this is not an equilibrium in the Nash sense. A player who knows or expects the other to volunteer with a high p_v_ might choose to defect for sure and thereby increase his or her payoff and reduce the other’s. In other words, using this ‘superrational’ strategy (Diekmann, 1985) requires the expectation that the other player will do the same.

### A Costly Error: The Over-Volunteering Hypothesis

Our third hypothesis is more subtle and thus riskier. We predict that many individuals will volunteer too much relative to formal standards and relative to the implications of their own expectations regarding others’ choices. They will, in other words, stumble into Popper’s dilemma of love. How might this happen? We submit that the social-distance heuristic is frugal in the sense that it has no non-monotonic provisos (Gigerenzer and Gaissmaier, 2011). There is no check as to whether there may be too much volunteering. Not having such a proviso works well in social dilemmas where mutual cooperation is the most efficient collective strategy (i.e., were 2R > T + P [or T + S]). In the VoD, however, heuristically thinking individuals may choose to volunteer for a very close other without working out the implications. As both individuals have this tendency, the outcome is inefficient. In other words, we predict that Archetti’s social preference model will offer a good description of volunteering over social distance, but that against this background of adaptiveness, there will be a systematic error precisely where individuals would want to avoid it the most.

When adding expectations to the picture, the possibility of over-volunteering becomes more poignant. If, as we hypothesize, people will be most likely to volunteer when the other is psychologically close, and if, as we also hypothesize, people project their own choices most strongly onto those who are close, then we will find that respondents over-volunteer even by the lights of their own expectations of reciprocity. To illustrate this hypothesis, imagine a pair of siblings. Both want to ‘do the right thing’ and sacrifice for the other. At the same time, they predict that their sibling is equally willing to make that sacrifice. Yet, they choose to volunteer. This outcome, if obtained, would suggest that projective predictions are difficult to alter. The player cannot escape the dilemma by defecting because this would suggest the worst personal and collective outcome. To avoid over-volunteering, the person would have to find a way to predict that the other person is less likely to volunteer than the self. This, in turn, might be a difficult psychological maneuver because it would suggest that the self is a more socially responsible person than the other. In doing so, it would undermine the perception of social closeness (there is, however, evidence for such self-enhancement in volunteering, Heck and Krueger, 2016, unpublished).

### Research Overview

We tested these hypotheses in three studies. In study 1, we sought to demonstrate the social-distance effect and provide evidence for over-volunteering at very short social distances, as evaluated against a game-theoretic standard. In study 2, we considered a full range of social distances and introduced respondents’ expectations. Here, we tested all three hypotheses (social projection, social distance, and over-volunteering) over multiple samples. In study 3, we manipulated expectations experimentally. Assuming that expectations are not epiphenomenal to behavior, we predicted that respondents would consult expectations when making a decision, but that the effect would be limited and result in over-volunteering.

## Study 1: Social Distance and Over-Volunteering

Undergraduate mostly female students (*N* = 250) in a 1st-year lecture course on social psychology at a German-language university in Switzerland took part in a classroom experiment. No demographic data were collected. Students received instructions over the microphone and were shown the following information on a large screen. Instructions read that “the goal of this experiment is to illustrate, with the help of your imagination, a social dilemma, that is a game for at least two persons, in which the consequences depend on the decisions of all participants. You will be asked to make a hypothetical decision that may entail that you or someone else will hypothetically receive an electric shock. Participation is anonymous and voluntary.” Next, participants were asked to imagine gradations of social distance using a method developed by Jones and Rachlin (2006) which asks participants to create a mental ranking of 100 people with rank #1 corresponding to a close friend or relative and rank #100 corresponding to a superficial acquaintance (see below for a detailed description of this method in the context of Study 2). Then the payoff matrix of the VoD was shown and explained. Students learned that they would receive 1 (hypothetical) electric shock if they volunteered, receive no shock if they did not volunteer while the other person did, and receive 2 shocks if neither they nor the other person volunteered. That is, the payoffs were *T* = 0, *R* = –1, and *P* = –2. This payoff structure is a simple linear transformation of the canonical structure discussed earlier and displayed in **Figure 1**. Using an online response interface, all participants made two binary decisions to either select Option A or Option B, which, respectively, amounted to volunteering and defecting. They made the first decision under the presumption that they were paired with the person of the lowest social distance (person #1 on the ranked list), and they made the second decision under the presumption of being paired with the person of the greatest social distance (person #100 on the list).

The results supported the social distance and the over-volunteering hypotheses. For the closest distance (rank #1), 87% volunteered. The 95% confidence interval, CI: [82; 91] excluded the equilibrium value of 75%, which would maximize joint outcomes. For the greatest distance (rank #100), 68% volunteered, and the 95% CI [62; 73] excluded its corresponding Nash equilibrium value of 50%, that is, the strategy of the rational, self-interested individual.

This was first evidence for the social distance hypothesis. Moreover, when compared with game-theoretic benchmarks, there was evidence for over-volunteering not only for a VoD involving close others but also involving distant others. Expectations were neither measured nor manipulated and no intermediate levels of social distance were considered. We designed a multi-sample study to address these issues.

## Study 2: A Continuum of Social Distance and Expectations

The goal of this study was to test the social distance, social projection, and over-volunteering hypotheses in the context of social expectations. We wanted to see whether people over-volunteer (at close distance) even in light of their own expecations regarding the other’s decision to volunteer. As discussed earlier, this prediction followed from the social projection hypothesis. In addition to tests of these three main hypotheses, the data also allowed us to ask whether respondents tended to think that they themselves were more likely to volunteer than others, and whether such a tendency might be moderated by social distance. If obtained, such a self-enhancement effect (“I volunteer more than the other”; Heck and Krueger, 2015) would constrain over-volunteering in the sense that it would make it less likely that people would volunteer with a high probability *and* expect the same from the other.

### Methods

#### Participants

We recruited a total of 703 participants in five samples, two of which came from a university campus in the Northeastern United States. Sample 1 was collected in the spring of 2013 and included 80 women and 80 men with a median age of 20 years. Sample 2 was collected in the spring of 2014 and included 94 women and 114 men with a median age of 20 years. Sample 3 was collected in the summer of 2014 at a campus in the German-speaking part of Switzerland. This sample included 62 men and 56 women (median age = 24). Samples 4 and 5 were collected in the fall of 2014 during a lecture class at the same Swiss University. Sample 4 (79 women and 26 men, median age = 21) received a dilemma with positive payoffs, whereas Sample 5 (76 women and 32 men, median age = 21) worked with negative payoffs (see below). Assignment to Samples 4 or 5 was random. All five samples shared nearly identical experimental procedures, which allowed us to analyze the data using a single statistical model in which the sample was entered as a potential moderator variable. This method offered an internal test of replicability and provided substantial statistical power (Schimmack, 2012). We describe the procedure for the largest sample (i.e., Sample 2) and note where the others differ.

#### Procedure

Participants were approached on an urban college campus in the Northeastern United States. All agreed to complete a brief survey on interdependent behavior. Each of 26 surveyors recruited eight respondents. The recruiters were enrolled in a laboratory course on social cognition, and they explained to the respondents that the data were being collected for a class project with the possibility of publication. Recruiters ensured that each respondent was surveyed individually and in a quiet location. The recruiter provided a sheet with instructions and the survey itself in a printed packet. The surveyor stayed on site, responded to questions of clarification, and thanked and debriefed the respondents upon completion of the survey.

The procedure for Sample 3 was slightly different in that only two surveyors recruited participants and no gender quota was used. For Sample 1, there were 20 surveyors. Samples 4 and 5 were collected during a lecture class with five teaching assistants distributing the questionnaires. Participants were promised a presentation on the results in return for their voluntary participation.

#### Materials

Instructions stated that the survey was designed “to tap into students’ intuitions regarding how they would behave in a situation in which they are interdependent with someone else. That is to say, what course of action would you choose if the outcome does not only depend on your choice but also someone else’s.”

The survey had three pages. On the first page, the VoD was described in neutral terms. Respondents were asked to “consider an interpersonal setting that is currently popular in studies on behavioral economics. The situation involves two individuals. Think of yourself as Person 1 and the other person as Person 2. Person 2 is anonymous with the exception of one bit of information, as you will see shortly. Both individuals must select a response at the same time and without knowledge of the other’s choice.” Next, the consequences of choosing Option A and Option B – by the respondent and the other person – were described. Mutual selection of Option A would result in 1 painful electric shock for each person and mutual selection of Option B would result in two painful shocks for each person. If one person selected Option A, while the other person selected Option B, the former would receive 1 shock, while the latter would receive none. This array of payoffs reflects the canonical volunteer’s dilemma; Option A amounts to volunteering, Option B to abstaining (see **Figure 1** for a normal form representation of the game and positive payoffs).

Next, the scale for social distance was introduced. Respondents read a modified version of Jones and Rachlin’s (2006) scale for the measurement of social distance. They were asked “to imagine that you have made a list of the 100 people closest to you in the world ranging from your dearest friend or relative at position #1 to a mere acquaintance at #100. The person at number one would be someone you know well and is your closest friend or relative. The person at #100 might be someone you recognize and encounter but perhaps you may not even know their name. You do not have to physically create the list—just imagine that you have done so.” Given this mental scale, respondents were asked to “consider five individuals from this hypothetical list (numbers 1, 25, 50, 75, and 100), and we will ask for two judgments in each case. Please note that we consider social distance to be symmetrical. However close or distant the other is to you, so you are to the other.”

The second page began with instructions of how to make probability judgments. To facilitate comprehension, the vivid language of frequencies was used. “In situations like the one we consider here, people might use different strategies. Suppose the game were played a 100 times; a person might decide to select Option A a certain number of times and Option B the rest of the times. This number, X out of 100, can represent the probability with which the person chooses Option A in a given individual situation.”

Roughly half of the respondents were first asked to provide judgments of the likelihood of their own choosing Option A, whereas the other half were first asked to judge the likelihood that the other person would choose Option A. Within each of these two counterbalanced conditions, roughly half of the respondents made ratings progressing from high to low social distance, whereas the remainder progressed in the opposite direction. These procedural variations did not have any effects on the response variables, nor did they moderate the effects of social distance. Thus, they were not further considered in Samples 4 and 5, in which we asked for the likelihood of their own choosing Option A first and used a low to high order for social distance.

The materials for Samples 3, 4, and 5 were exact translations of the materials for Sample 2. The main differences between materials for Sample 1 and Sample 2 were that (a) the cooperative response option was labeled “Volunteer” and the other option was labeled “Abstain” for Sample 1, whereas the neutral labels “Option A” and “Option B” were used for Sample 2, and (b) the instructions for the probability judgment were more ambiguous for Sample 1 in that participants were asked “How certain are you that you would volunteer (vs. abstain)? Write in a percentage value between 0 and 100.” A final difference was that the scenario described in Sample 4 was not about an electric shock, but about pleasant electrical stimulation. For example, participants were told that if they chose Option A and the other player chose Option B, they would receive one pleasant electrical stimulation and the other player would receive two pleasant electrical stimulations.

To check comprehension, we asked participants in Sample 3 at the very end to go back to the probability of volunteering they had stated for a randomly selected level of social distance, and indicate the most likely outcome of a single game based on their probability of volunteering and their expected probability of the other player volunteering. Five options were given, namely the four outcomes defined by the payoff matrix and all outcomes equally probable. Due to an oversight we did not include the case in which two of the outcomes would be most probable (which would arise if either own probability of volunteering or expectation was equal to 0.5). This led to ambiguities for 9 out of 117 participants (8%) who correctly selected one of the two most probable outcomes. By treating these participants separately, we estimate the level of comprehension conservatively. The results reassured us that participants generally understood the game. Correct answers were given by 73 participants (62%); 33 participants (28%) gave wrong answers, and 2 participants did not answer the question.

### Results

#### Analyses

**FIGURE 2:**
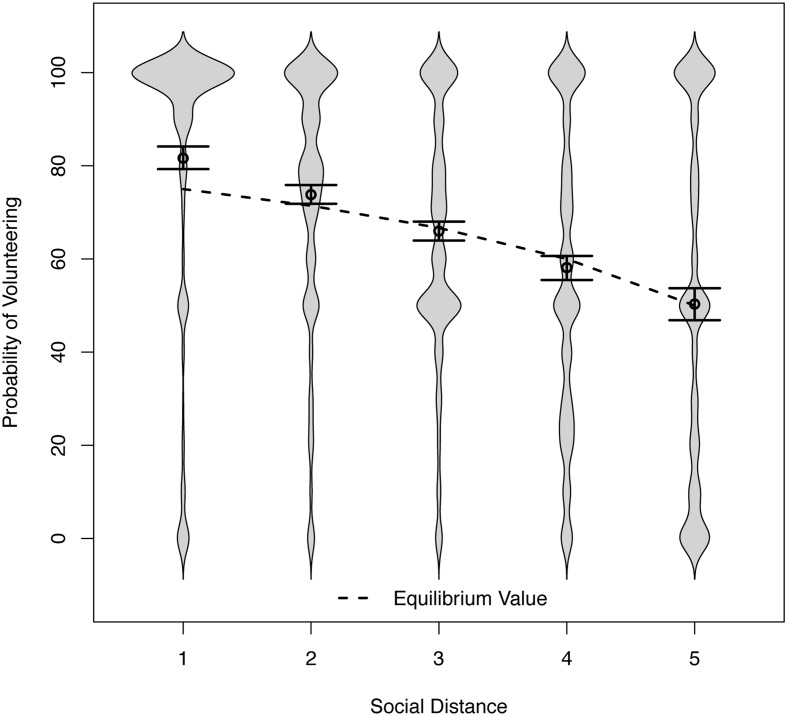
**Distributions of the probability of volunteering across social distance conditions in Study 2 (and 95% confidence intervals for the means).** The lower limit of the confidence interval for the second lowest social distance condition (71.84) excludes the equilibrium value (71.43).

Preliminary analyses revealed homogeneous results with the exception of Sample 4, where outcomes were framed as gains. We continue with analyses of the negative-frame VoD and return to the findings from Sample 4 later. **Figure 2** displays the distributions of volunteering as bean plots, with their widths reflecting the density of responses (Kampstra, 2008) at specific levels of social distance. To account for the skew in the data, we estimated standard errors and confidence intervals by bootstrapping. We modeled heterogeneity in the average levels of the response variables and the effects of social distance as random effects, using linear mixed models algorithms provided by the package lme4 (Bates et al., 2014) for the software R (R Core Team, 2014). To obtain standardized effect sizes, we used a function provided by LaHuis et al. (2014) which calculates the approximate explained variance at Level 1.

#### The Probability of Volunteering

The means shown in **Figure 2** (circles) support the social distance hypothesis. Volunteering (choosing ‘Option A’) became less likely as social distance increased. To model this trend, we regressed the stated probability of volunteering on social distance (coded from 1 = lowest distance, to 5 = highest distance). To account for differences between samples, we used unweighted effects coding with three indicator variables and their interactions with the social distance variable. The intercepts and the effect of social distance represent the unweighted mean intercept and slope, respectively, for the whole dataset (i.e., all samples except for Sample 4, see below).

The intercept of the regression was *b* = 89.47 and the slope was *b* = -7.83, with a 95% confidence interval (CI) [-8.92, -6.69]. With each stepwise increase in social distance, the reported probability of volunteering decreased by 7.83 percentage points. The approximate explained variance at Level 1 was *R*^2^ = 24%. The individual sample intercepts and slopes from the different samples were not significantly different from the overall intercept or slope (all |*t*| s < 1.49), which permits a joint analysis of the data.

Further analysis revealed that almost all respondents became less willing to volunteer as social distance increased. Only a few individuals produced curvilinear patterns or positive regression weights (such that the higher the social distance, the greater the stated probability of volunteering). We will return to this group when we examine the relationship between expectations and volunteering.

**Figure 2** also shows the game-theoretic benchmarks for the probability of volunteering as a dotted line (Archetti, 2009). These theoretical values fit the empirical data well. There is, however, one noteworthy exception, and it corroborates the hypothesis of over-volunteering. At the two shortest social distances, respondents volunteered with a probability greater than the probability that would maximize joint outcomes (if used by both players). This mean-level difference underestimates the prevalence of over-volunteering because of the skew in the distribution. To understand how a randomly selected individual participant would choose, the width of the beans provides better guidance. For low social distance, the beans vividly illustrate the excess prosociality. In the lowest and second-lowest social distance conditions, 78 and 65% were over-volunteers, respectively (i.e., volunteering with a probability greater than the equilibrium value). The corresponding figures for those who volunteered with certainty were 59 and 31%.

#### Expectations of Other’s Volunteering

**FIGURE 3:**
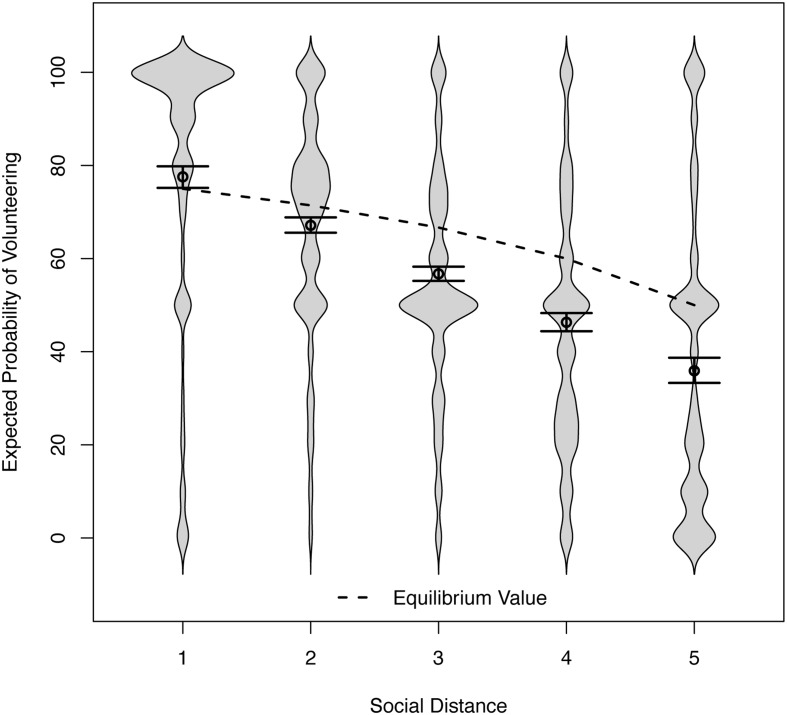
**Distributions of the expected probability of volunteering across social distance conditions in Study 2 (and 95% confidence intervals for the means).** For shortest social distance, the equilibrium value (75) is below the lower limit of the confidence interval (75.19).

We predicted that expectations regarding the other’s probability of volunteering would also decrease over social distance, and would thus be correlated with one’s own probability of volunteering. **Figure 3** shows that the data supported this prediction. In a regression of expectation on social distance, the intercept was *b* = 87.97 and the slope was *b* = -10.42, with a 95% CI [-11.41, -9.32]. The approximate explained variance at Level 1 was *R*^2^ = 36%. Expected volunteering deteriorated over social distance faster than own volunteering did, thereby linking the size of the self-enhancement bias to social distance. In all but the smallest social distance conditions, respondents expected the other player to volunteer with a probability below the equilibrium. Conversely, for the closest other person, they expected others to volunteer above the equilibrium value. In other words, respondents expected the closest other player to volunteer with a greater probability than would be optimal for the dyad, mirroring the results obtained for their own volunteering. The implication is that respondents were willing to volunteer for close others with a probability that was too high in light of their own high expectations of those others volunteering.

#### The Relationship between Volunteering and Expectations

We tested the social projection hypothesis by regressing own volunteering on expected volunteering in a mixed model with random intercepts. As predicted, the slope of this regression was positive (*b* = 0.55, intercept = 34.95). The approximate explained variance at Level 1 was *R*^2^ = 34%. Even when considering only the data of the few participants who volunteered with a higher probability as social distance increased (*n* = 85; 14%), the slope was positive (*b* = 0.20, 95% CI [0.10, 0.29]). For these individuals, the association between behavior and expectation was weaker (*p* < 0.01) than for the majority (*b* = 0.61; 95% CI [0.58, 0.64]). The respective values of approximate explained variance at Level 1 were *R*^2^ = 1% for the subset of participants with positive slopes and *R*^2^ = 44% for the majority. This is strong support for the projection hypothesis. No matter which way respondents changed their willingness to volunteer over social distance, they expected others to do the same. Yet, the minority of respondents showing a positive distance effect may have had a poorer understanding of the game. In Sample 3, 73% of the participants with a negative slope for the social distance effect passed the comprehension check, whereas only 55% of participants with a positive slope did.

We assume that the correlation between own willingness to volunteer and the volunteering expected from others arises from processes of social projection rather than “introjective” mechanisms that align one’s own decision with what is expected of others. It is difficult to imagine how expectations might arise without reference to one’s own behavioral inclination. Indeed, if it were possible to construct such expectations early and independently, then one’s own decision should be positively matched with the expected behavior of the other only when social distance is short; when individuals are paired with strangers, that is, when they act only in their own self-interest, they should do the opposite of what they expect the other to do. Yet, within each level of social distance, we find positive associations between behavior and expectation. When regressing expectations on decisions, the slope was steepest for the shortest distance (*b* = 0.55; 95% CI [0.49, 0.61]); *R*^2^ = 28%), but it was positive for the remaining four levels too (overall *b* = 0.46; 95% CI [0.42, 0.49]; *R*^2^ = 22%).

#### Positive Outcomes

We returned to the data obtained in Sample 4, in which payoffs were positive. Here, the slope of the regression of volunteering on social distance was flatter than it was for negative outcomes (*b* = -6.40, 95% CI [-9.12, -3.33]; *R*^2^ = 14%) and the intercept lower (79.85). As a result, the mean value of volunteering at the shortest social distance was 73.44, and the 95% CI [65.01, 81.07] included the equilibrium value (75).

We obtained similar results for the expectations regarding the probability of the other player volunteering in that the slope was flatter and the intercept lower compared with the results for negative outcomes (*b* = -6.69, 95% CI [-9.56, -3.54]; *R*^2^ = 14%; intercept *b* = 77.53). The mean value of expected probability of the other player volunteering at the shortest distance was 70.84 and the 95% CI [62.66, 78.39] included the equilibrium value (75).

For the positive outcomes too, expectations predicted volunteering (*b* = 0.84, intercept *b* = 12.11). With an approximate explained variance at Level 1 of *R*^2^ = 73%, this effect was much stronger than for negative outcomes. Within levels of social distance, own decisions predicted expectations well, and this relationship was again strongest when distance was short (*b* = 0.99 and 0.89 for the first two levels and 0.70 thereafter with respective values of explained variance *R*^2^ = 86, 77, and 61%). Again, the findings suggest that participants made their own decisions to volunteer by consulting the available payoffs and weighting them by social distance, and then assuming that others would do the same.

### Discussion

The results of this multi-sample study supported the main hypotheses. In support of the social-projection hypothesis, we found positive correlations between respondents’ willingness to volunteer and their predictions of what the other person would do. These correlations emerged for each level of social distance, and they were strongest for short distances. It is worth noting that some “differential projection” (Robbins and Krueger, 2005), that is, a decrease of perceived similarities over social distance, is warranted because actual similarities also tend to decrease. Closely related and connected individuals share more similarities than do mere strangers. Expecting such similarities in behavior from one another is therefore a generally adaptive strategy.

As predicted, the willingness to volunteer and correspondent expectations both decreased over social distance, thereby allowing errors of over-volunteering to creep in. For the two shortest social distances, willingness to volunteer exceeded game-theoretic benchmarks. While this result suggests over-volunteering, it is not yet definitive. Respondents might rationally exceed these benchmarks if they (have reason to) believe that the others are less likely to volunteer. The clearest case for over-volunteering requires that both, own willingness to volunteer and others’ expected willingness to volunteer, lie above the benchmark. We find such evidence for the shortest social distance.

Given the moral overtones of volunteering, we predicted and found evidence of self-enhancement. At each level of social distance, respondents claimed that they were, on average, more willing to volunteer than the other person. The self-enhancement bias is not a striking discovery on its own, but it is relevant in that it makes over-volunteering more difficult to detect. Had self-enhancement been any stronger, volunteers would have expected others to defect, in which case they would have expected successful (anti-)coordination to the benefit of the other.

Following theory and research on social projection, we submit that people construct expectations about others on the basis of their own behaviors rather than *vice versa* (see Van Veelen et al., 2016, for a comprehensive review of the evidence for this claim and its boundary conditions). This causal flow has good support in research on both social projection and self-enhancement (Krueger, 2007; Heck and Krueger, 2015). Yet, it is difficult to draw firm inferences in the VoD because, as in other social dilemmas, decisions and expectations are dynamically interdependent. To open a window into the potential role of expectations on volunteering decisions, we manipulated expectations in our final study. Induced expectations are available before respondents make strategic decisions (Gaschler et al., 2014). This design let us test two hypotheses: First, expectations will inversely affect volunteering decisions. Second, the effect of expectations will be smaller than full rationality demands. A consequence of this underuse of expectations is over-volunteering. Respondents will be willing to volunteer even when they expect the other person to volunteer as well.

## Study 3: The Causal Effect of Expectation

We tested these hypotheses in a two-factorial repeated-measures design, in which the social distance between the respondent and the other person was either very low or very high, and in which the respondent was either led to believe that the other person was very likely or very unlikely to volunteer. Besides anticipating a replication of the social distance effect, we predicted that respondents would be more willing to defect when the other was likely to volunteer than if the other was unlikely to volunteer. In other words, we predicted an effect of expectation contravening the direction seen in the two correlational studies. We had no reason to think that social distance would moderate the size of this effect. A subtler and riskier prediction was that the expectation effect would be smaller than required by expected-value considerations. We induced expectations so strong that a strictly value-maximizing person would either defect (if expectation of other’s volunteering is high) or volunteer (if expectation is low). We doubted that these floors and ceilings would be empirically matched in size. Critically, we predicted that the shortfall relative to the floor of no volunteering would be greater than the shortfall relative to the ceiling of full volunteering. Such an asymmetry would constitute evidence of over-volunteering.

### Method

We recruited 296 residents of the United States on Amazon Mechanical Turk and collected no further demographic information. Each participant received a small payment of c75 and a lottery ticket for a $25 Amazon.com gift card. Each participant responded to all four scenarios of the 2 (social distance: high vs. low) by 2 (expectation: high vs. low) design.^3^

The structure of the VoD and the social distance scale were introduced as in the previous studies, using a standard platform (Qualtrics Research Suite [Survey software], 2014). Participants were asked to consider only the closest (distance rank 1) and the remotest person as a partner in the VoD (social distance rank 100). For each dilemma, they were to assume either that this person was very likely to volunteer (with a 80% chance) or very unlikely to volunteer (20% chance). The order of the four scenarios was randomized over participants in a 2 (distant or closest partner first) × 2 (for the first partner: high or low expectations first) × 2 (for the second partner: high or low expectations first) design. Participants then entered their own likelihood to volunteer using a percentage scale.

### Results and Discussion

**FIGURE 4:**
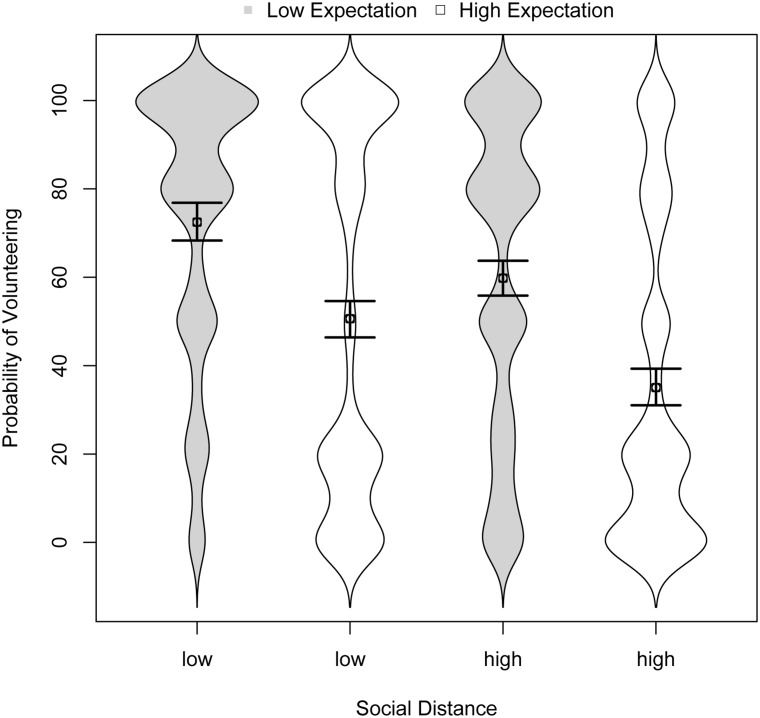
**Distributions of volunteering across conditions in Study 3 (and 95% confidence intervals for the means)**.

**Figure 4** shows the findings as bean plots with means and confidence intervals. Visual inspection reveals clear evidence for both the social distance hypothesis and the expectation hypothesis. We again used linear mixed models with random effects and bootstrapped confidence intervals for statistical analysis and effect-coding (-0.5 and 0.5) for the predictor variables social distance and expectation. The main effect of social distance, *b* = -14.13, 95% CI [-10.84, -17.54], *R*^2^ = 3%, indicated that participants were approximately 14% less likely to volunteer for the distant other compared with the close other. The main effect of expectation, *b* = -23.30, 95% CI [-18.52, -28.08], *R*^2^ = 9%, indicated that participants were about 23% less likely to volunteer when they expected the other to volunteer with a probability of 80% vs. 20%. The interaction term was not significant, *b* = -2.92, 95% CI [-8.64, 2.72], *R*^2^ < 0.1%.

The data also support the over-volunteering hypothesis. When the other was expected to volunteer with an 80% probability, the optimal response was to not volunteer at all. Yet, participants announced that they would volunteer with a 51 and 35% probability, respectively, for the close and distant other (see **Figure 4**). This is *prima facie* evidence for over-volunteering. Yet, there was also the converse effect of undervolunteering when the other was expected to volunteer with a 20% probability. Although the optimal response was to volunteer with certainty, participants announced that they would volunteer with a 72 and 60% probability, respectively, for the close and distant other.

The results of Study 3 replicate and extend the body of correlational findings accumulated in Studies 1 and 2. The social distance effect on volunteering is robust, consistent with the ideas of inclusive fitness (Hamilton, 1964) and strong reciprocity (Gintis, 2000). As the social distance heuristic uses a single cue, it opens the door to predictable error. We have identified over-volunteering as one such an error and we saw that respondents violate their own expectations regarding the choices of others when they arguably care the most about an efficient outcome. Study 3 shows that this violation of expectation occurs not only when these expectations are self-generated but also when they are externally provided.

## General Discussion

### Summary and Review

Volunteer’s Dilemmas pervade social life, although they are rarely recognized as such. Who will buy the wine for dinner? Who will start work on the co-authored manuscript? Who will punish the loafers and jaded bystanders (Przepiorka and Diekmann, 2013)? The VoD has received little research attention apart from the specific issue of bystander intervention and apathy in emergency situations (Darley and Latané, 1968; Krueger and Massey, 2009; Fischer et al., 2011). We suspect that the VoD is neglected because of the belief that it is easily resolved with a little goodwill and coordination, particularly among kin and the well-acquainted (Sir Karl Popper dissenting). Most research remains focused on social cooperation in public-goods and resource dilemmas involving unrelated strangers (Dawes, 1980; Norenzayan et al., 2016). In those dilemmas, collective outcomes continue to improve as more individuals contribute. In contrast, the relationship between collective welfare and the frequency of prosocial behavior is non-linear in the VoD. It is inefficient to have more than one volunteer or to have none at all. This non-linearity poses a psychological challenge. A prosocial person must consider the risk of making a redundant and thus inefficient contribution.^4^

An excess of prosociality can occur when individuals are close and when the effects of volunteering or mutual failure to volunteer are negative. Our principal explanation of this finding is the idea that people use a social-distance heuristic when deciding whether to accept the cost of volunteering. They are willing to make a sacrifice to the extent that the other person is socially, psychologically, or genetically close to them. This heuristic works well in many contexts of interdependence, providing adaptive advantages that are recognized by evolutionary biologists and game theorists (e.g., Ferrière and Michod, 2011; Locey et al., 2013). Indeed, we find that the mean probability of volunteering tracks the predictions of a formal equilibrium model, which uses relatedness to weight and integrate the other person’s outcomes with one’s own (Archetti, 2009). When social distance is zero, the model assumes that players care for the outcomes of the other player as much as they do for their own.

Our findings suggest that many pairs of close individuals will end up with the same outcome, the R payoff for mutual volunteering, although they would have fared better if their probability of volunteering had been lower. It is not clear yet whether this effect is large enough so that individuals can gain insight into its non-optimality. Perhaps they will focus instead on the equality of their two payoffs, consider it fair, and find reassurance in the successful avoidance of the most aversive outcome of mutual defection (Leliveld et al., 2009). Alternatively, our findings point toward a mistaken sense of altruism (Krueger, 2011; Oakley et al., 2011), which, under certain conditions, can do great harm. For instance, when individual and group identities fuse, the eagerness to act prosocially can beget tragedy (Whitehouse et al., 2014).

Now consider the relevance of the findings regarding expectations of volunteering. With pain at stake, people expect close others to volunteer, and even over-volunteer. Why do respondents not scale back their own probability of volunteering to restore maximum efficiency? The logic of social projection suggests an answer (Krueger, 2013). Consider a person who is ready to volunteer and who expects others to do the same. This person cannot switch from ‘volunteer’ to ‘defect’ without assuming that others will do the same. If projection is a valid heuristic for inferring the actions of others, it is valid regardless of one’s particular strategy. Like prosocial behavior, social projection decreases over social distance (Robbins and Krueger, 2005); this general finding emerges in the present data too (Studies 1 and 2) and thus helps explain the tenacity of over-volunteering among close individuals.

If – as we believe – respondents generated their expectations about the likely behavior of others after they had made their own decisions, we can make sense of a final finding: respondents thought that the probability of others to volunteer was lower than their own. With volunteering being a socially desirable act, declaring oneself to be more willing to volunteer than others amounts to a better-than-average-effect (Alicke and Sedikides, 2009). Self-enhancers claim dual moral credit (Heck and Krueger, 2016). They not only volunteer but also predict that they volunteer more than others do. Self-enhancement is consistent with the general projective pattern (Heck and Krueger, 2015). If respondents derive expectations about others from their own decisions, these expectations should be more regressive (i.e., less extreme) than own decisions (Moore and Healy, 2008). Indeed, expectations were overall closer to the 50% mark than were judgments of own intended volunteering.

In light of the bounded rationality with which people approach the VoD, we may ask what options exist for efficient solutions. In contrast to the prisoner’s dilemma and the assurance game, but like the game of chicken (Van Lange et al., 2014), the VoD yields best results if the two players act differently. Over repeated encounters, turn-taking in volunteering yields mutual benefits. In a one-shot episode, however, communication is of little help. If both individuals declare their intention to volunteer (or defect), additional factors must be brought in to break the tie. One reasonable social rule is to put the burden of volunteering on whomever can afford it the most (Przepiorka and Diekmann, 2013). When Linda and Laura reach for the lunch bill, jobless Linda may yield to working Laura (Abele et al., 2014). When there is no difference in wealth, timing is critical. Whoever announces their decision first forces the other to do the opposite (Schelling, 1960). We suspect that in such a sequential arrangement social distance will remain a moderating factor.

### Open Questions

Our study designs reflect choices made under constraints and in the interest of expediency. Future research needs to identify and test pinpoint hypotheses to sharpen our theoretical understanding of the volunteer’s dilemma and to enhance the generalizability of the findings.

First, there is the finding that over-volunteering occurred only for aversive outcomes. It may be too soon to declare valence a robust moderator as we had only one sample with a positive game frame. If, however, the valence effect survives further testing, we may note that the departure from rationality and adpativeness occurred where participants would arguably be most motivated to avoid it: in the domain of pain (Kahneman and Tversky, 1984; Baumeister et al., 2001).

Second, the task of mapping the effects of social distance onto the predictions of a rational equilibrium model limited us to a artificial methodology. To scale social distance with precision, we sacrificed the real-life experience of encountering others in the dilemma. As future research meets the challenge of mundane realism, it will be critical to remain wary of confounds. Individuated partners will introduce a host of additional information or assumptions that might increase the variability of results in random or systematic ways.

Third, the use of five levels of social distance presented in non-random order may raise the specter of experimental demand. Yet, we remain sanguine because the demand hypothesis makes no specific predictions. What particular slope or which specific intercept, for example, should a respondent feel called upon to produce when scaling her own willingness to volunteer onto social distance?

Fourth, we presented the VoD as a choice problem of the type used in scenario research in the psychology of judgment and decision-making (see, for example, Murnighan et al., 1993, or Kim and Murnighan, 1997, for such work on the VoD). In contrast, behavioral economics prizes consumable payoffs. Recent work in our laboratory suggests that in the VoD, symbolic payoffs yield the same results as material ones do (Krueger et al., 2016, unpublished).

Many ordinary people and the scientists who study them operate from the simple, reasonable, and adaptive heuristic that prosocial behavior is socially desirable. Their moral concerns take the form of asking what can be done to make such behavior more common. Our excursion into the volunteer’s dilemma suggests structural and psychological factors can combine to undercut the effects of good intentions and expectations. More is not always better.

## Ethics Statement

The studies were exempt. Survey research with no conceivable risk to participants.

## Author Contributions

JK conceived the project, supervised data collection, consulted with data analysis, and drafted the manuscript. JU conceived the project, supervised data collection, consulted with data analysis, and drafted the manuscript. LC conceived the third experiment, collected and analyzed data, and helped with manuscript preparation.

## Conflict of Interest Statement

The authors declare that the research was conducted in the absence of any commercial or financial relationships that could be construed as a potential conflict of interest.

## Footnotes

1 If there is support for the projection hypothesis in the VoD, we will have an argument against the idea that people project strongly in the PD only *in order to rationalize their own cooperative desires.*

2 This prediction refers to group averages. It is conceivable to find strict defectors whose primary goal is to exploit the other, or, equivalently, to ensure not to earn a lower payoff than the other.

3 An additional manipulation asked respondents to either seek to maximize their own payoffs or to maximize the joined payoffs. This manipulation had no effect on the results and is henceforth ignored.

4 The VoD is akin to a step-level public-good dilemma, in which a benefit is provided to all once a threshold of contributive cooperation is reached. All additional contributions are wasted.
